# Four-Chamber Intracardiac Thrombi Complicating Wild-Type TTR Amyloidosis

**DOI:** 10.1155/2018/1845962

**Published:** 2018-12-20

**Authors:** Jan M. Griffin, Daniel P. Judge, Kenton J. Zehr, Jose Madrazo, Rosanne Rouf

**Affiliations:** ^1^Division of Cardiology, Department of Medicine, Johns Hopkins Hospital, Baltimore, Maryland, USA; ^2^Department of Cardiothoracic Surgery, Johns Hopkins School of Medicine, Baltimore, Maryland, USA

## Abstract

Cardiac amyloidosis is a rare disease, and its prevalence varies depending on the type of amyloid protein involved. Several case reports make reference to the increased risk of thrombosis and thromboembolic events in cardiac amyloidosis. We report a case of rapidly evolving, multichamber thrombi in a patient who was ultimately diagnosed with wild-type TTR cardiac amyloidosis.

## 1. Background

Cardiac amyloidosis results from the abnormal extracellular deposition of amyloid protein in the heart, particularly light chain (AL), wild-type transthyretin (ATTRwt), or familial transthyretin (ATTRmut) amyloid [1]. The type of amyloid protein involved determines the patient's clinical course and treatment options. Several case reports make reference to the increased risk of thrombosis and thromboembolic events in cardiac amyloidosis [2, 3]. We report a case of rapidly evolving, multichamber thrombi in a patient who was ultimately diagnosed with ATTRwt cardiac amyloidosis.

## 2. Case Presentation

A 73-year-old African American male presented to an outside hospital with a three-week history of shortness of breath on exertion, 3 pillow orthopnea, and bilateral lower extremity swelling. The patient had been diagnosed with heart failure with reduced ejection fraction (HFrEF) 4 months prior, but he stopped his cardiac medications when he developed worsening symptoms. Other medical history included hypertension, diabetes mellitus, hyperlipidemia, and ongoing high alcohol consumption. Physical examination revealed bilateral lower extremity edema, elevated jugular venous pressure, and bibasilar pulmonary rales. One day after admission, his troponin I peaked at 18 ng/ml (reference range < 0.04 ng/ml). Electrocardiography revealed nonspecific T wave changes in the anterolateral leads. He was treated for acute coronary syndrome (ACS) with aspirin, clopidogrel, low molecular weight heparin, and intravenous furosemide. A transthoracic echocardiogram (TTE) showed an ejection fraction (EF) of 30-35%, moderate biatrial enlargement, and moderate right ventricular (RV) systolic dysfunction. In addition, a 2.0 cm spherical mobile echodensity was noted to be attached to the left side of the interatrial septum, concerning for myxoma vs. thrombus. Given the TTE findings and the need for coronary angiography, transfer was requested to our hospital.

**Figure 1:**
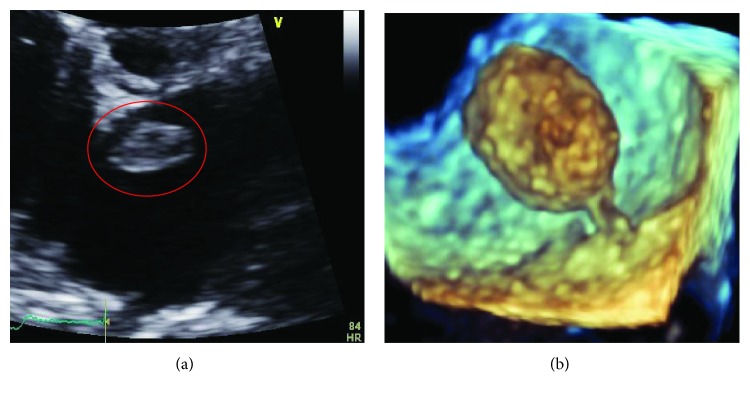
Transthoracic parasternal short axis (a) and transesophageal 3-D (b) images of pedunculated left atrial mass.

**Figure 2:**
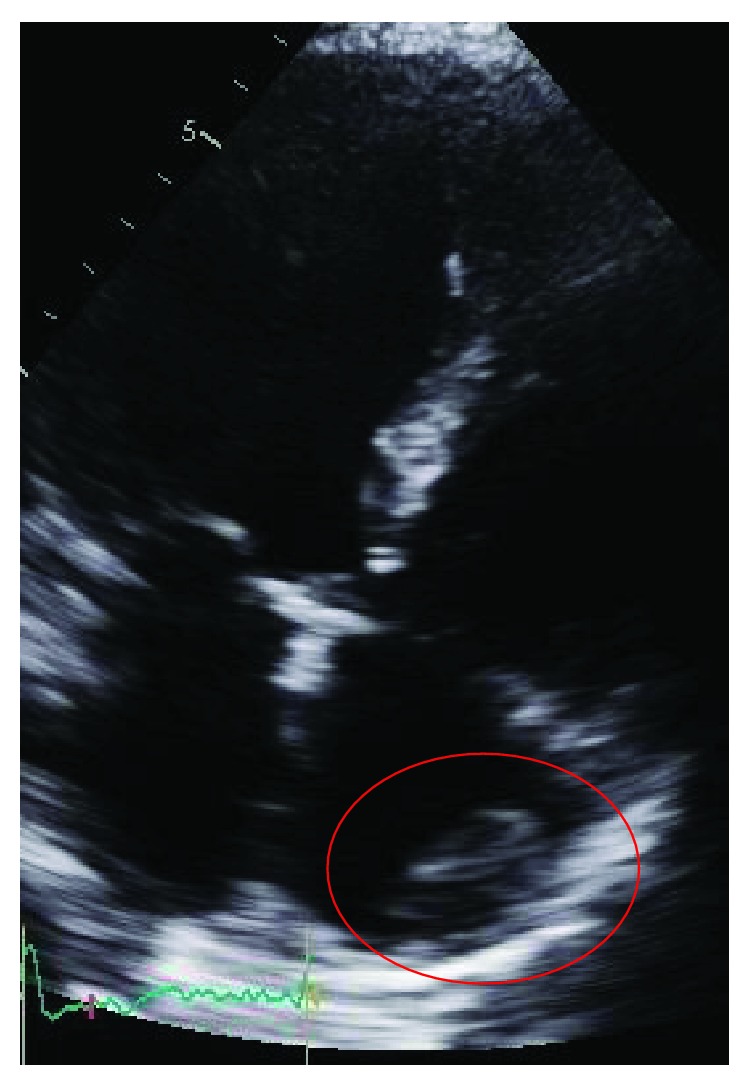
Right atrial thrombus (TTE inverted 4-chamber view).

**Figure 3:**
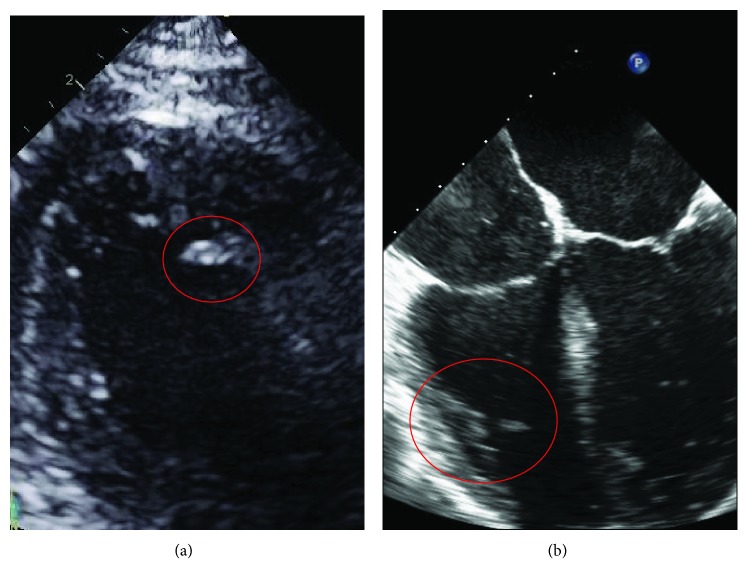
Zoomed-in transthoracic apical 4-chamber view of left ventricular (a) and transesophageal 4-chamber view of right ventricular (b) thrombi.

**Figure 4:**
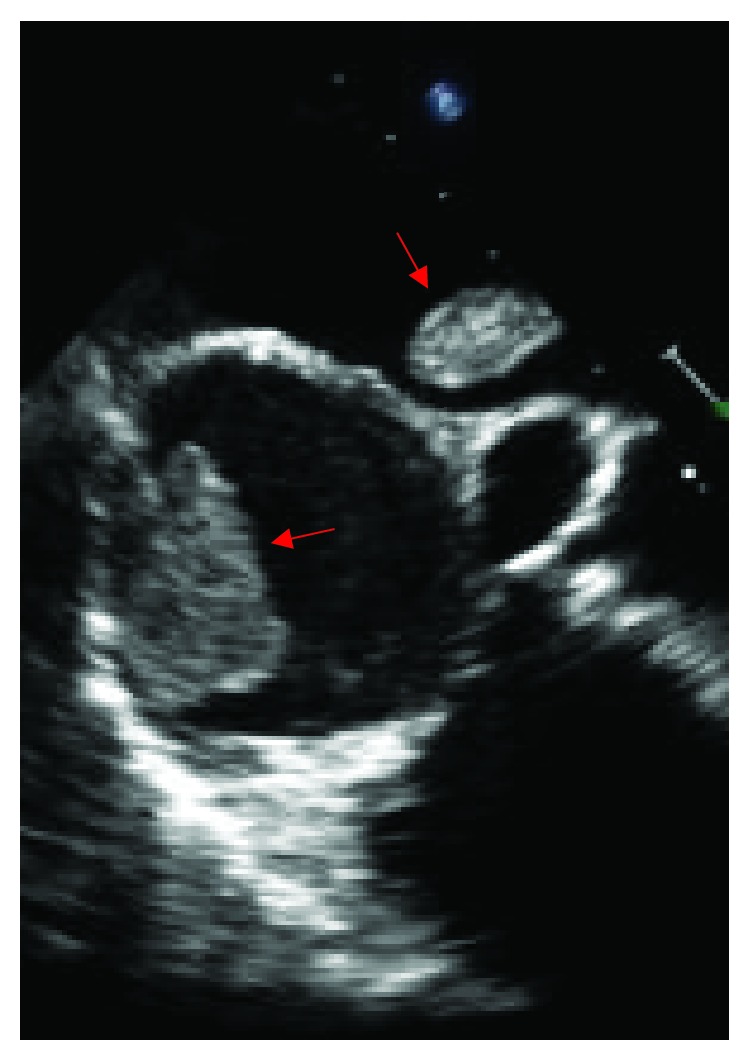
Zoomed-in transesophageal echocardiogram 4-chamber view of left and right atrial thrombi.

Coronary angiography was performed and showed thrombotic subtotal occlusion of the ramus intermedius coronary artery branch, thought likely to be the culprit lesion. He also had 80-90% occlusion of the second obtuse marginal (OM2) and 50% occlusion of the distal left anterior descending (LAD) artery. Due to the need for further evaluation of the left atrial (LA) mass, no intervention was performed. TTE subsequently showed severe global hypokinesis of the left ventricle (LV) (EF 10-15%) and mild LV hypertrophy. The previously noted LA mass measured 2.1 cm by 1.4 cm, appeared pedunculated and was attached to the left side of the interatrial septum with echogenic characteristics concerning for atrial myxoma (Figure 1). Within the right atrium (RA) was a large (3.2 × 2.0 cm) semimobile mural thrombus (Figure 2), and spontaneous echo contrast was also evident in the LV cavity. He remained anticoagulated with unfractionated heparin. Despite this, subsequent TTE imaging demonstrated the development of biventricular thrombi over the course of several days (Figure 3). On hospital day 7, he went to the operating room. Intraoperative transesophageal echocardiogram (TEE) showed a new subcentimeter mass on the RV free wall. The previously noted masses in the RA and LA were resected (Figure 4). Two-vessel coronary artery bypass grafting was performed with the left internal mammary artery to LAD and saphenous vein graft to OM2. Given that the etiology of his cardiomyopathy was also yet to be determined, an intraoperative LV biopsy was performed. Pathology of both the RA and pedunculated LA masses showed organized thrombus. The LV biopsy was negative for histopathologic evidence of inflammatory infiltrate, granulomas, or eosinophils but was strikingly positive for amyloid deposition by Congo red staining. Liquid chromatography tandem mass spectrometry demonstrated ATTRwt amyloidosis and did not detect an amino acid sequence abnormality in the TTR protein. The patient's postoperative recovery was complicated by a left frontal cerebrovascular accident, thought likely to be cardioembolic in nature. He was discharged for rehabilitation on postoperative day 15 on chronic anticoagulation with warfarin.

## 3. Discussion

We attribute this patient's rapidly evolving four-chamber intracardiac thrombi to his underlying ATTRwt amyloid cardiomyopathy and concomitant systolic dysfunction. To our knowledge, this is the first report of thrombi identified in all four cardiac chambers in a patient with cardiac amyloidosis. A previously published report suggests one case of multichamber thrombi wherein biatrial thrombosis resulted in death by massive pulmonary embolus [2]. Intriguingly, a study of autopsy patients from the Mayo Clinic involving 116 cardiac amyloid cases (mainly AL and ATTRwt) found intracardiac thrombi in 33% (38) of hearts [4], most of which had a single thrombus. Fifteen of 116 cases had between 2 and 5 thrombi, and the majority of which were in the RA or the LA with 23 embolic events identified, 19 of which were fatal [4]. Another study [5] of 156 patients with mainly AL and ATTRwt cardiac amyloidosis reported that 27% had one or more thrombi, with a prevalence of 18% in ATTRwt, though all occurred in the setting of concurrent atrial fibrillation. Related risk factors for intracardiac thrombosis for our patient were intermittent atrial fibrillation during hospitalization, depressed EF from occlusive coronary artery disease, and possible alcohol cardiomyopathy.

It has been shown that the intracardiac thrombosis in ATTRwt or ATTRmut amyloid is associated with more advanced amyloid deposition [4]. The mechanism for thrombus formation in cardiac amyloid is not known. Amyloid infiltration may play a role in mechanical dysfunction, which contributes to blood stasis, thus promoting clot formation even in sinus rhythm [4, 6]. Endomyocardial and endothelial damage secondary to amyloid deposition [7, 8] and a hypercoagulable state [9] are also thought to be involved. In cardiac amyloidosis, anticoagulation is indicated for patients with atrial fibrillation, regardless of their CHA2DS2-VASc score [10]. Intriguingly, our patient continued to have thrombus formation while adequately anticoagulated; however, a full hypercoagulability evaluation could not be performed while on therapeutic anticoagulation, and so we cannot rule out a concomitant hypercoagulable state. It is also interesting that this patient evolved into having intracardiac thrombi in all four chambers.

In summary, embolization of the LA or LV thrombus into the ramus intermedius coronary artery likely precipitated ACS and reduced EF and the subsequent diagnosis of cardiac amyloidosis in this patient. Epicardial LV biopsy revealed ATTRwt amyloid on histopathology. This case emphasizes the need for a high suspicion for cardiac amyloidosis in patients with intracardiac thrombosis and LV dysfunction.
